# Performance of a point-of-care ultrasound platform for artificial intelligence-enabled assessment of pulmonary B-lines

**DOI:** 10.1186/s12947-025-00338-2

**Published:** 2025-03-03

**Authors:** Ashkan Labaf, Linda Åhman-Persson, Leo Silvén Husu, J. Gustav Smith, Annika Ingvarsson, Anna Werther Evaldsson

**Affiliations:** 1 Department of Clinical Sciences Lund, Cardiology, Section for Heart Failure and Valvular Disease, Lund University, Skåne University Hospital, Klinikgatan 15, Lund, 221 85 Sweden; 2 Department of Internal and Emergency Medicine, Skåne University Hospital, Malmö, Sweden; 3 Department of Molecular and Clinical Medicine, Institute of Medicine, University of Gothenburg, Gothenburg, Sweden

**Keywords:** Lung ultrasound, B-lines, Artificial intelligence, POCUS

## Abstract

**Background:**

The incorporation of artificial intelligence (AI) into point-of-care ultrasound (POCUS) platforms has rapidly increased. The number of B-lines present on lung ultrasound (LUS) serve as a useful tool for the assessment of pulmonary congestion. Interpretation, however, requires experience and therefore AI automation has been pursued. This study aimed to test the agreement between the AI software embedded in a major vendor POCUS system and visual expert assessment.

**Methods:**

This single-center prospective study included 55 patients hospitalized for various respiratory symptoms, predominantly acutely decompensated heart failure. A 12-zone protocol was used. Two experts in LUS independently categorized B-lines into 0, 1–2, 3–4, and ≥ 5. The intraclass correlation coefficient (ICC) was used to determine agreement.

**Results:**

A total of 672 LUS zones were obtained, with 584 (87%) eligible for analysis. Compared with expert reviewers, the AI significantly overcounted number of B-lines per patient (23.5 vs. 2.8, *p* < 0.001). A greater proportion of zones with > 5 B-lines was found by the AI than by the reviewers (38% vs. 4%, *p* < 0.001). The ICC between the AI and reviewers was 0.28 for the total sum of B-lines and 0.37 for the zone-by-zone method. The interreviewer agreement was excellent, with ICCs of 0.92 and 0.91, respectively.

**Conclusion:**

This study demonstrated excellent interrater reliability of B-line counts from experts but poor agreement with the AI software embedded in a major vendor system, primarily due to overcounting. Our findings indicate that further development is needed to increase the accuracy of AI tools in LUS.

**Supplementary Information:**

The online version contains supplementary material available at 10.1186/s12947-025-00338-2.

## Introduction

The European Society of Cardiology guidelines for heart failure currently recommend the use of chest X-ray and lung ultrasound (LUS) to confirm heart failure and investigate other potential causes of breathlessness [1]. LUS is an excellent tool for assessing pulmonary edema and interstitial lung syndrome (ILS) by detecting the presence of B-lines. Multiple studies have demonstrated that LUS is superior to chest X-ray in evaluating extravascular lung water (EVLW) [2–4]. LUS is also highly accessible and more easily implemented since it can be performed at the bedside. Additionally, there are significant prognostic implications for discharged patients with residual pulmonary congestion [5], a condition that is difficult to identify on the basis solely of clinical status and patient weight. Therefore, there is growing support for tailoring the diuretic therapy on the basis of LUS findings aimed at reducing the risk of rehospitalization [6], especially if this approach can be applied more objectively and standardized.

A central component in LUS is the detection of B-lines, which are defined as vertical bands of hyperechoic laser-like artifacts originating from the pleural line that transverse the total sector without fading and occur with ILS [7, 8]. The number and morphology of B-lines have been shown to be correlated with the amount of EVLW [9], however, they are not specific to cardiogenic pulmonary oedema [8]. Although the detection and assessment of B-lines requires experience, high inter- and -intraobserver agreement among experts has been demonstrated in COVID-19 [10] and ILS [11] patients. This makes the assessment suitable for standardization, potentially with the assistance of artificial intelligence (AI) embedded in the software of various ultrasound vendors. The software utilized in this study is part of the globally leading ultrasound platform [12], offering real-time B-line counting and aiming to improve inter- and intraobserver reliability between examinations, even when conducted by less experienced examiners.

The aim of this study was to evaluate the accuracy of AI software from a major ultrasound vendor in detecting B-lines via a 12-zone protocol in a heterogeneous, real-world cohort, including patients with heart failure, and to validate these results against expert assessments.

## Methods

### Patient selection

This was a single-center prospective study conducted during 2023 at one tertiary university hospital in Sweden. Adult patients (> 18 years) hospitalized at the Internal Medicine Department, including the day care unit, and at the Cardiology Department at the University Hospital of Skåne were recruited for the study. The patients were being predominantly treated for various respiratory symptoms, with acutely decompensated heart failure being the most common main diagnosis. The participants had to understand the purpose of the study and be able to provide informed consent. Additionally, they needed to be able to sit in an upright position. Patients with known lung disease or pneumonia were excluded, except for those with pulmonary embolism and asthma.

### Study setting

**Fig. 1:**
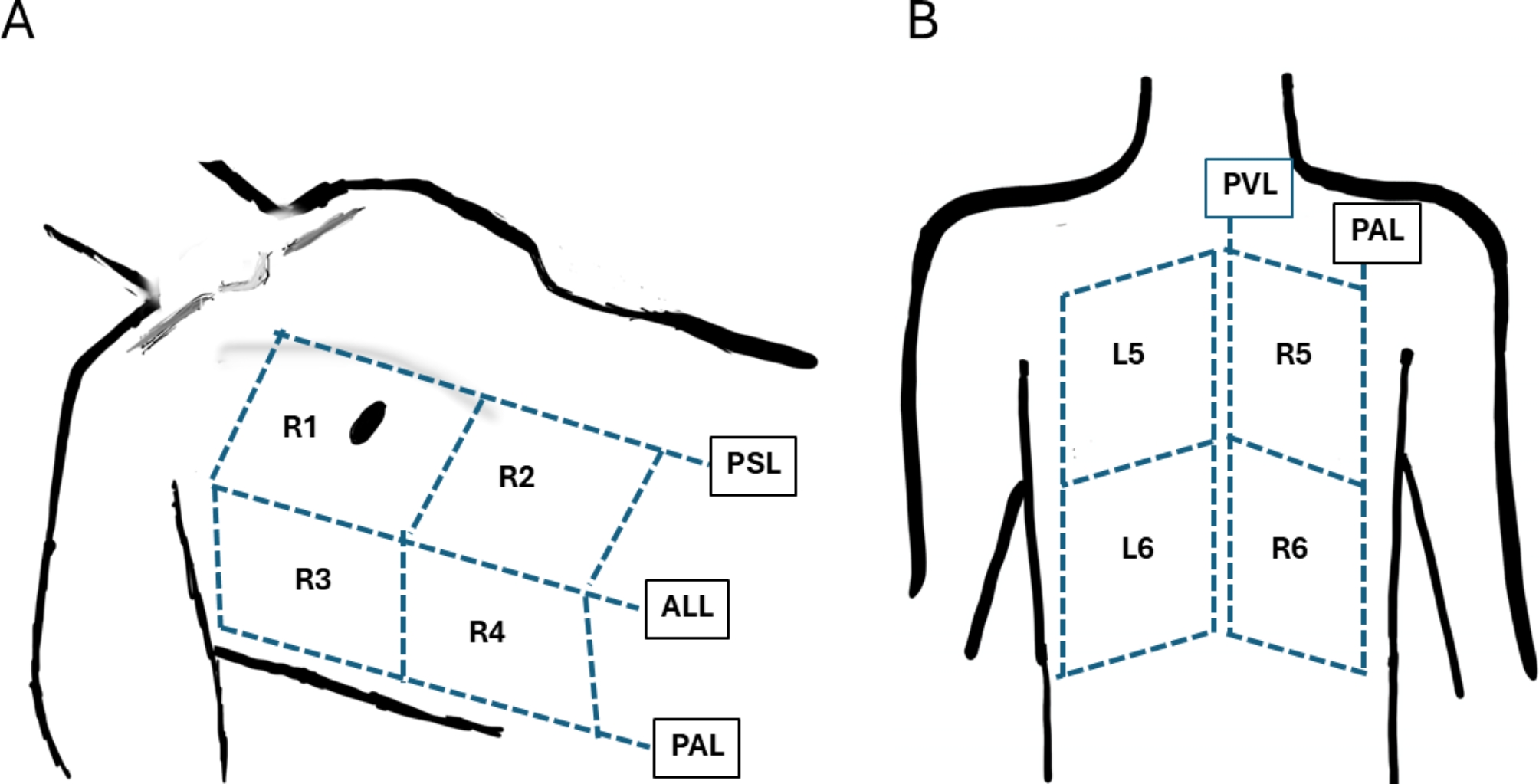
Scanning zones in the 12-zone protocol. (**A**) The anterior zones are divided into four regions on each hemithorax, delineated by the parasternal line (PSL), anterior axillary line (AAL), and posterior axillary line (PAL). (**B**) The posterior zones are divided by the paravertebral line (PVL) and the PAL, comprising a total of six zones per hemithorax

The included patients were examined bedside using a Venue GO ultrasound system (General Electric Healthcare, Wauwatosa, WI, USA) with a curved (C1-5) low-frequency (1.5–5.7 MHz) probe set to an imaging depth of 16 cm. Each patient was examined in a total of 12 zones, according to the zones in Fig. 1. Initially, the patient was placed in a supine position with the bed inclined at 45 degrees, and 4 zones were recorded on each anterolateral side of the thorax. The zones were divided horizontally by the parasternal line, the anterior axillary line, and the posterior axillary line. The anterior chest wall extended from the sternum to the anterior axillary line and was further divided into upper and lower halves. The lateral zone spans from the anterior to the posterior axillary line and was also divided into upper and basal halves. The patient subsequently seated in an upright position at the side of the bed, and 2 zones on each side of the back were recorded. The dorsal zones were divided by the height of the tip of the scapula, creating the posterior superior and posterior inferior zones. The transducer was held in the sagittal plane between two rib spaces, and recordings lasted 4–5 s per zone. All examinations were performed by one examinator to reduce the interrater reliability.

**Fig. 2:**
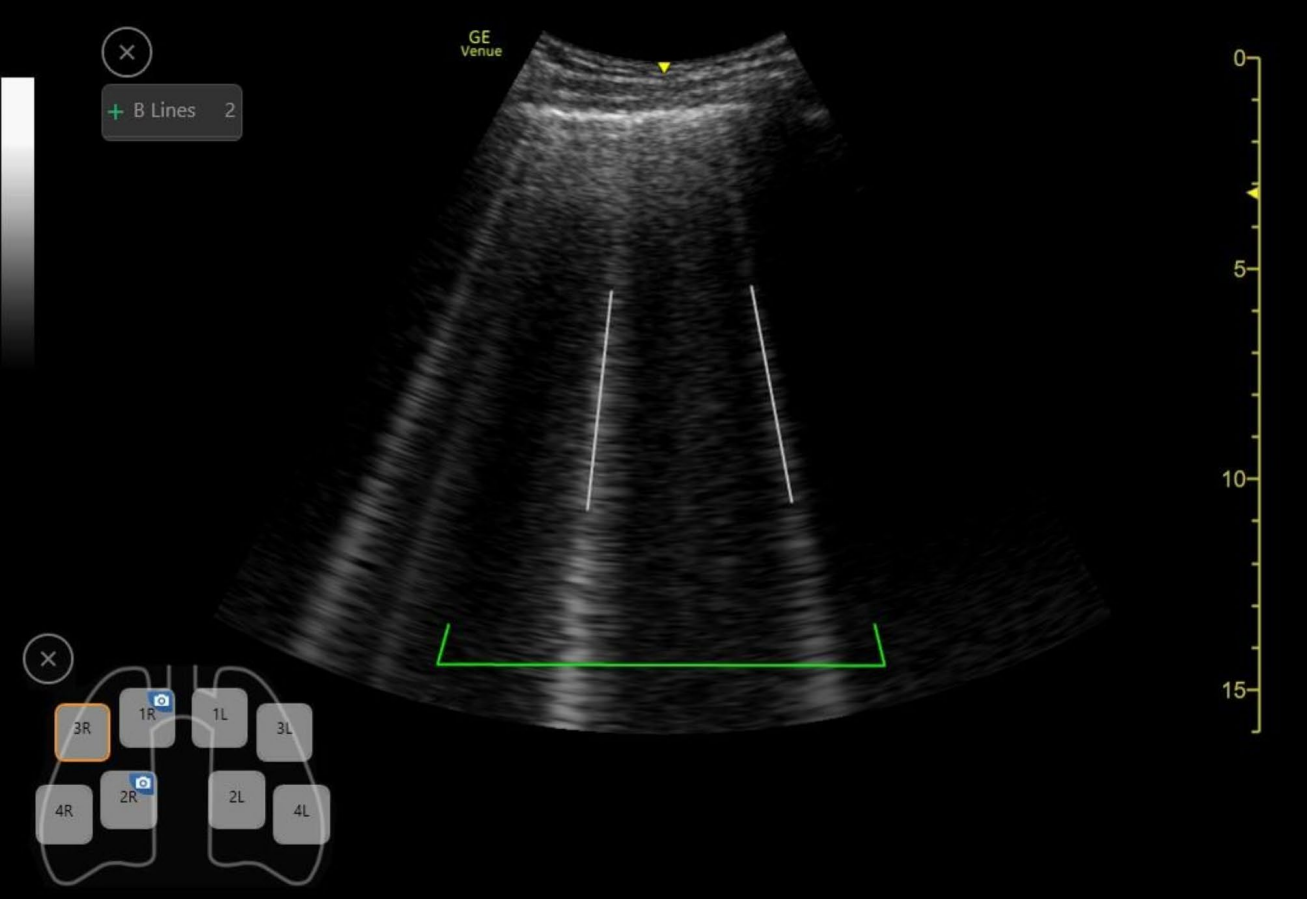
The auto B-line tool counts B-lines in real-time and highlights the frame with the highest B-line count

The auto B-line tool determines the overall lung score by identifying and quantifying B-lines in real time, emphasizing them visually, and showcasing the image with the maximum B-line count (Fig. 2). At the end of the scan, the tool calculates the total B-line count by selecting the frame with the highest number of B-lines displayed within a pictogram for each zone. The auto B-line tool was utilized in all recordings, categorizing the B-lines into 0, 1, 2, 3, 4, and > 5 for each zone. Zones that were not appropriate for assessment due to pleural effusion, nonvisible pleura, or suboptimal recording quality were excluded from the B-line correlation analysis but are presented in the results. The AI results were blinded, and two experts in LUS interpretation independently categorized the B-lines for each zone into 0, 1–2, 3–4, and ≥5. To identify potential misinterpretations caused by the AI tool, all zones where the AI tool detected the ≥1 B-line were carefully reviewed to determine the type of misinterpretation, if any. These were subsequently categorized as follows: evaluating the adjacent rib space with or without B-lines, misinterpreting shred lines, or assessing the rib shadow adjacent to the center of the screen. The ultrasound expert physicians had extensive experience in ultrasound. One is a cardiologist with expertise in cardiac ultrasound with experience in LUS at a tertiary center, while the other is an internal medicine consultant with experience in LUS, the author of the region-wide applicable guidelines for LUS and the ultrasound director for the POCUS-academy, a hospital-wide organization for training and clinical use of POCUS. The examinator was an internal medicine resident in training for POCUS. In conjunction to LUS, the examinator also conducted a clinical evaluation of each patient’s volume status.

This study was approved by the Regional Ethics Review Board of Lund, EPN. Written informed consent was obtained from all patients.

### Statistics

Categorical variables are expressed as frequencies (percentages) and continuous variables are expressed as the means ± standard deviations or medians (interquartile range). The experts classified the B-lines into categories of 0, 1–2, 3–4, and > 5, which differed from the categorization of the AI software, as described above. For the analysis, B-lines detected by the AI software were adjusted to 1.5 for cases categorized as 1 or 2, and 3.5 for those categorized as 3 or 4, to align the comparisons more closely. The number of B-lines was compared zone-by-zone, as was the total sum for each patient. To assess interrater agreement, an intraclass correlation coefficient (ICC) analysis was performed. The targets were treated as random effects, while the raters were considered fixed effects. A two-way mixed model was used to compare the total B-line count between the algorithm and the experts, measuring absolute agreement for correlation. A 95% confidence interval (CI) was applied to the analysis. The ICC was analyzed for the total sum of B-lines as well as on a zone-by-zone basis. Furthermore, Bland-Altman analysis was used to assess the agreement between the AI counts and expert counts; mean value of the two measurements [13]. All analyses were performed using SPSS 29 (IBM. Inc., USA).

## Results

In total, 672 LUS zones were obtained from 55 patients. The baseline characteristics and diagnoses are presented in Table 1. The most common diagnosis was congestive heart failure (35%). The median B-line count and B-line distribution among the reviewers and AI are presented in Table 2. There was a significant difference in the B-line count between the reviewers (2.8) and the AI (23.5), *p* < 0.001. A high proportion of B-lines > 5 (38%) was found by the AI compared with the reviewers (4%), with significant differences between the groups (*p* < 0.001).

**Table 1:** Patient characteristics of the study population

Patient characteristics	*N* = 55
Age (y, mean SD range)	77 ± 13
Male	36 (66%)
Peripheral status	
*Euvolemic*	33 (60%)
*Hypervolemic*	22 (40%)
Diagnosis	
*Heart failure* *Atrial fibrillation* *Chest pain*	19 (35%) 6 (11%) 4 (7%)
*Dyspnea*	4 (7%)
*Infection*	4 (7%)
*PE/DVT* *Kidney failure* *Syncope*	2 (4%) 2 (4%) 2 (4%)
*Other*	12 (22%)

**Table 2:** Distribution of total B-lines by method of quantification and median B-line count for all participants

Method of quantification		Median B-line count per patient (Q1:Q3)		B-line distribution (% of sequences). *n* = 584				
				0 B-lines		1–2 B-lines	3–4 B-lines	≥ 5 B-lines
Mean expert visual agreement		2.8 (0:8.5)		457 (80%)		66 (12%)	29 (5%)	22 (4%)
AI count		23.5 (17.7:31.0)		277 (48%)		14 (2%)	64 (11%)	219 (38%)

Q1:Q3 – first and third quartile; n – total number of sequences

Among the 672 recorded clips, 574 (85%) were used for the analysis. Among the excluded clips, 11 (1.6%) were assessed as being of insufficient quality for evaluation, 26 (3.9%) did not properly identify the pleural line, and 51 (7.6%) exhibited pleural effusion. Among the 26 clips without an identifiable pleural line, 22 (85%) were in the Zone 2 L, where the presence of the heart obscured the lung sector. Pleural effusions were predominantly found in the posterior inferior zones, 32 (63%), and lateral zones, 15 (29%).

The total sum of B-lines assessed by the reviewers was significantly greater in hypervolemic patients than in euvolemic patients, 4.6 and 0.75 respectively (*p* = 0.04).

The mean score for each lung zone, as evaluated by both the reviewers and the AI, is presented in Table 3, along with the corresponding ICC. There was a slight tendency for higher B-line counts in the R6 and L6 zones across all assessments, and the ICC values varied and were generally low among the different zones. All zones where the AI assessed ≥1 B-line were reviewed by examining the loops to identify where potential B-lines were detected. This review resulted in three categories. Among the 574 zones assessed by the AI, 81 (14%) were misinterpreted when the adjacent rib space was evaluated, with or without B-lines. There were 6 zones (1%) that involved subpleural consolidations with shred lines (C-lines), and in another 6 zones (1%), the AI misinterpreted rib shadows between two intercostal spaces as B-lines.

**Table 3:** Mean of the zones and ICC

Lung zone	Reviewer 1	Reviewer 2	AI	*ICC (95% CI)
R1	0.32	0.27	1.83	0.31 (-0.11-0.59)
R2	0.40	0.19	2.90	0.17 (-0.17-0.46)
R3	0.59	0.47	2.45	0.35 (-0.11-0.63)
R4	0.64	0.49	2.40	0.37 (-0.13-0.66)
R5	0.27	0.28	2.43	0.17 (-0.17-0.45)
R6	0.93	0.78	2.38	0.63 (0.06–0.84)
L1	0.65	0.46	2.04	0.50 (0.04–0.73)
L2	0.40	0.12	2.56	0.23 (-0.26-0.59)
L3	0.67	0.63	2.09	0.55 (0.09–0.76)
L4	0.78	0.75	2.20	0.50 (-0.17-0.75)
L5	0.26	0.16	2.53	0.18 (-0.17-0.47)
L6	0.91	0.77	2.77	0.48 (-0.10-0.75)

*ICC between the mean of the reviewers and AI

The agreement between the reviewers was high for both the total sum method and the zone-by-zone method, indicating excellent interreviewer agreement, as presented in Table 4. The correlation between the reviewers and the AI was slightly greater when the zone-by-zone method was used than when the total sum method was used; however, the overall correlation coefficients were poor.

**Table 4:** Intraclass correlation between expert agreement and AI tool

Method of quantification, total sum	ICC	95% CI
Between reviewers	0.92	0 0.86– 0.96
AI and reviewers	0.28	-0.12–0.64

**Method** **of quantification**,** zone-by-zone**	**ICC**	**95% CI**
Between reviewers	0.91	0.89– 0.93
AI and reviewers	0.37	-0.01–0.59

**Fig. 3:**
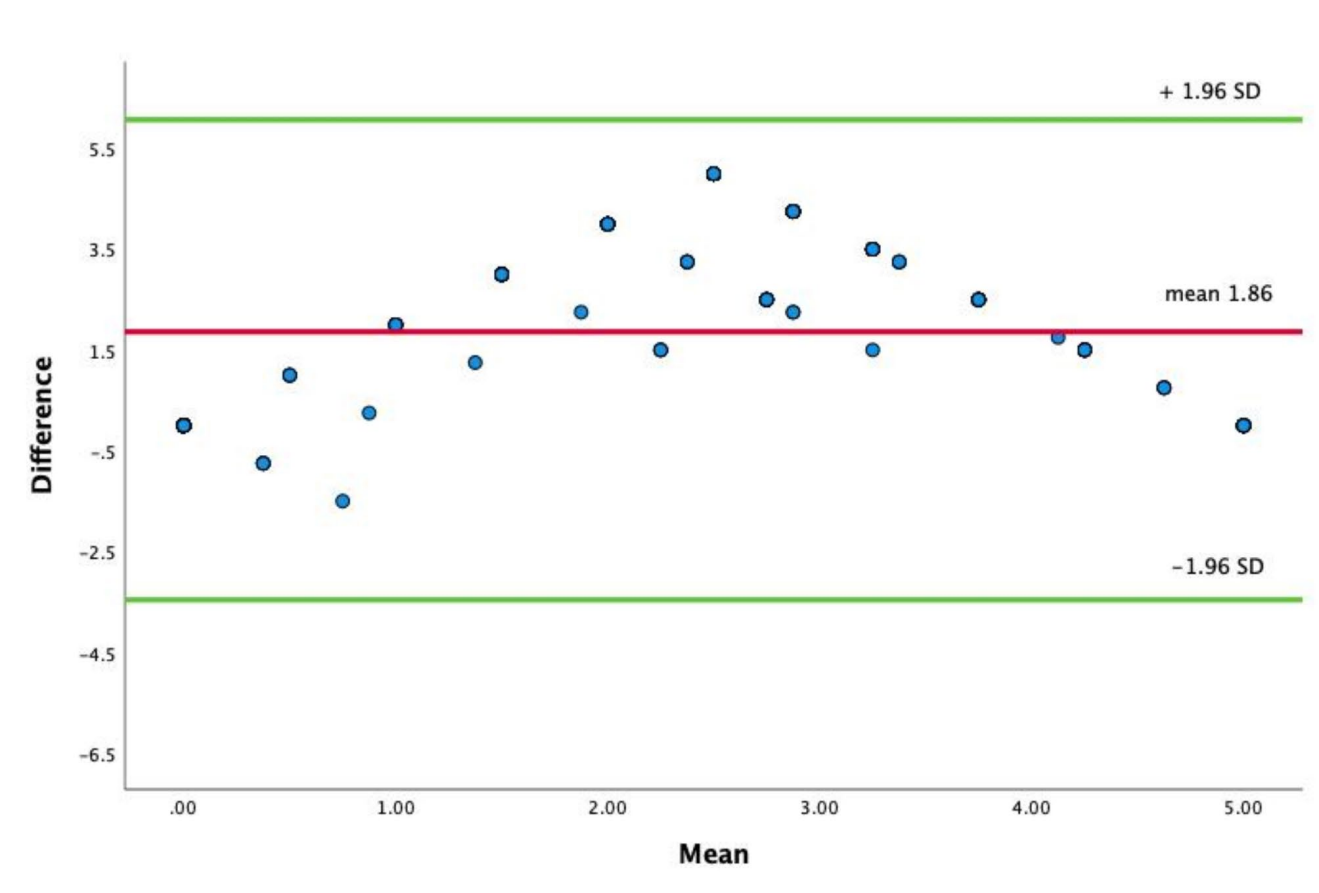
Bland-Altman Plot of the reviewers and the AI. The x-axis displays the average B-lines for both the AI and the reviewers, while the y-axis shows the mean difference between the measurements. The red line indicates the average difference between the two measurements (1.86), with the upper and lower lines representing the 95% limits of agreement between the two measurement methods

A Bland-Altman Plot was generated for the reviewers and the AI, as presented in Fig. 3. The mean of the difference and bias was 1.86 ± 2.15, and the limits of agreement were − 2.35 and 6.07, respectively. The Bland-Altman plot shows that a significant number of scans have a high positive difference, with a mean of 2.5.

## Discussion

In this study, we compared the B-line counts determined by an AI-software embedded in a commercially available ultrasound device to those assessed by LUS expert physicians. The comparison revealed a poor correlation between the AI and the expert evaluations, both in terms of the total sum and with regard to the zone-by-zone method. However, the interreviewer agreement was excellent. LUS image acquisition aids in the confirmation of congestive heart failure as well as the detection of residual lung congestion and AI has the potential to facilitate the management of patients with heart failure and help reduce the interoperator variability. Nevertheless, our results suggest that further development is needed in the algorithm of the automated tool to increase its correlation with expert assessments before it can be considered reliable for clinical decision making.

The total sum of B-lines per patient differed widely between the assessments. Overall, the AI tended to overcount B-lines throughout all the lung zones. The anterior (zone 2) and posterior superior (zone 5) zones exhibited the lowest correlation, where the overcounting occurred the most. The heart being in view in the left anterior zones can presumably interfere with image quality and affect the AI assessment. Interestingly, the lateral and posterior inferior zones exhibited greater correlation, even though lateral image acquisition is typically more challenging to obtain. Another observation was that the AI predominantly categorized B-lines as either 0 (48%) or ≥ 5 (38%), with very few assessments falling between 0 and 5 (13%). It appears that when the AI detects B-lines, there is a tendency to overestimate their quantity. The Bland-Altman plot indicates that many zones were assessed as having 5 B-lines by the AI, whereas the visual assessment indicated 0 B-lines. The cause of the overcounting is not entirely clear, but several factors could be involved. B-lines can appear in various lung pathologies and conditions. During the SARS-CoV-2 pandemic, an expert consensus document was published that proposed a distinction between B-lines and comet tail artifacts [14]. True B-lines are associated with a smooth pleural reflection caused by cardiogenic pulmonary edema and present in a diffuse pattern. In contrast, comet tail artifacts appear in various lung disorders with irregular, fragmented pleural reflections, which can be focal or diffuse and may vary in width, resembling a comet with a narrow head and a wide tail. One possible explanation for our findings is that the AI software may struggle to differentiate between these two entities. Furthermore, our review revealed that 14% of the B-lines identified by the AI were in fact based on evaluation of the adjacent rib space, often without any B-lines present in those adjacent spaces. This occurred despite the examinations being conducted with the intercostal space centrally positioned and utilizing the prespecified lung setting on the machine.

Another important consideration is that, given the variety of conditions in our cohort, some patients had lung consolidations that presented with irregular boundaries, often resembling fractal lines. These boundaries, separating consolidated lung tissue from the underlying aerated lung, can produce shred signs or C-lines, which might be misinterpreted by the software as true B-lines. Our review revealed that this misinterpretation occurred in 1% of the B-lines assessed by the AI.

Previous studies reported better agreement between AI and experts’ visual assessment. Short et al. [15], investigated the reliability of automated B-lines with the same software used in our study, which included only four patients, but demonstrated good to excellent interobserver reliability (ICC 0.79). This study has been utilized as a reference in the marketing of the AI-embedded software. Russel et al. [16], assessed novice learners’ ability to obtain LUS images with AI quantifying B-lines via these images, showing a fair correlation when compared with that of an expert reviewer, who used the same AI software as that used in our study. However, the ICC between the AI and expert was only 0.56. Furthermore, Damodaran et al. [17] studied B-lines in COVID-19 patients and compared the visual assessment with AI via software, the results revealed similar ICCs of 0.52-0-53. These figures indicate barely a moderate correlation and more closely align with our findings than the reference study from Short et al. However, numerous neural network and deep learning algorithms have been developed recently, demonstrating reliable performance in quantifying B-lines, albeit with limitations such as a small number of patients and potential bias associated with industry-sponsored studies [18, 19]. A key distinction in our study is the use of a 12-zone protocol, which specifically included posterior zones, with patients positioned upright. However, a recent study showed that a 2-zone anterior-superior thoracic ultrasound protocol can provide similar information to an 8-zone approach in datasets of patients with heart failure [20]. 

AI approaches can be divided and categorized into different methodologies. Initial efforts often focus on visual perceptions with prespecified algorithms. However, the future probably lies in leveraging large datasets and employing deep learning techniques in conjunction with expert visual assessments to increase accuracy and reliability. In line with this, recently a deep learning AI model was developed that demonstrated a strong correlation with expert-level B-line quantification in detecting B-lines, while outperforming a group of operators with varying levels of experience [19]. The quantification of B-lines in patients with heart failure is likely to become even more prevalent in the future because of its bedside applicability, speed, and non-radiation nature. Therefore, this field is only at its beginning, and AI tools for quantification of B-lines will develop into more accurate systems becoming a certainty in everyday practice.

Our study has several limitations that need to be highlighted. First, the study cohort could have been larger to obtain more accurate correlations, even though 672 zones were scanned. Second, only one examiner was used to obtain the images which may have influenced the quality of both the AI and visual expert counting. Third, we used a heterogeneous cohort of patients to mimic the real-world practice, making it difficult to compare our findings with those of other studies that included more homogenous cohorts. Fourth, the AI software had the ability to count only 0–4 and ≥5 B-lines per lung zone. Although counting more than 5 B-lines in one sector becomes less relevant and is sufficient to label it as an ILS, this limitation affects the overall correlation analysis.

## Conclusion

Our study demonstrated that the AI-software imbedded in a major vendor system is poorly correlated with visual expert agreement when B-lines are counted. While the interrater reliability was excellent, the AI tended to overcount B-lines across all the lung zones. This suggests that further development is needed to improve the accuracy of the AI tool, and that caution should be exercised when AI is used for B-line assessment in clinical practice.

## Electronic supplementary material

Below is the link to the electronic supplementary material.

Supplementary Material 1

## Data Availability

No datasets were generated or analysed during the current study.
