# Supplementary material for: Performance of a point-of-care ultrasound platform for artificial intelligence-enabled assessment of pulmonary B-lines
Source: Cardiovasc Ultrasound. 2025 Mar 3;23:3. doi: 10.1186/s12947-025-00338-2 (PMC11874383; doi:10.1186/s12947-025-00338-2)
Supplement: Supplementary file 1 — Supplementary Material 1 [file 12947_2025_338_MOESM1_ESM.pdf]

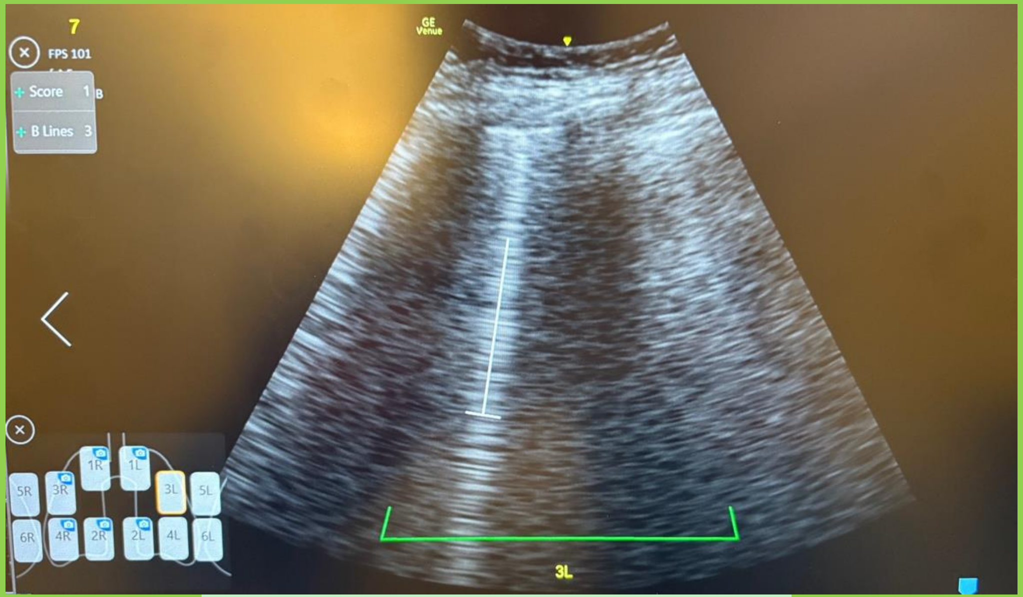

AI-generated B-line counts

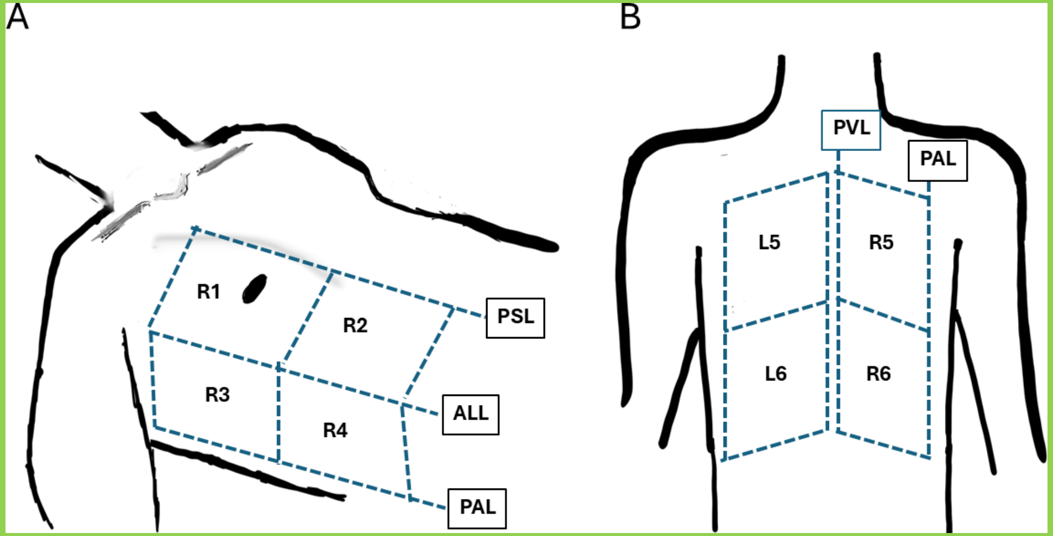

12 zone lung ultrasound protocol

Performance of POCUS platform for AI enabled assessment of pulmonary B-lines

- Single center study with 55 patients using a 12 zone lung ultrasound protocol
- An automatic B-line tool in a major vendor was utilized
- AI B-line counts were compared to two expert reviewers using intraclass correlation coefficient (ICC)

ICC between AI and experts

0.28

ICC between experts

0.92
